# Safety and efficacy of direct oral anticoagulants in bioprosthetic valves: A systematic review and meta-analysis

**DOI:** 10.3389/fcvm.2023.1099591

**Published:** 2023-02-27

**Authors:** Lubna Bakr, Ahmed Elsayed, Omar Saleh, Mostafa Abdalraouf, Ghulam André Ng, Mokhtar Ibrahim

**Affiliations:** ^1^The Royal College of Surgeons of England, London, United Kingdom; ^2^Liverpool University Hospitals NHS Foundation Trust, Liverpool, United Kingdom; ^3^NHS, Health Education England – North West (HEENW), Manchester, United Kingdom; ^4^Suez Canal University Hospital, Ismailia, Egypt; ^5^Sheikh Zayed Specialized Hospital, Giza, Egypt; ^6^Glenfield Hospital, University Hospitals of Leicester NHS Trust (UHL), Leicester, United Kingdom; ^7^Department of Cardiovascular Sciences, University of Leicester, National Institute for Health Research Leicester Biomedical Research Center, Leicester, United Kingdom; ^8^Department of Cardiology, Ain Shams University, Cairo, Egypt

**Keywords:** direct oral anticoagulants, vitamin K antagonists, transcatheter aortic valve implantation, bioprosthetic valves, atrial fibrillation, meta-analysis

## Abstract

**Background:**

Direct oral anticoagulants are efficient alternatives to vitamin K antagonists. There is little evidence regarding their use in patients who underwent bioprosthetic valve replacement whether surgically or through a transcatheter approach and have another indication of anticoagulation. Trials have compared different members of the DOACs family to VKAs and showed that they were at least non-inferior to VKAs with regard to safety and efficacy. However, this is still controversial. Our meta-analysis aims at providing a clearer view of their future use in this subgroup of patients.

**Methods:**

PubMed and Cochrane were searched for randomised clinical trials and observational studies. Bleeding, stroke, and all-cause mortality were the outcomes of interest.

**Results:**

Ten papers with a total of 4,088 patients were included. Our meta-analysis revealed no significant differences between the incidence of bleeding between DOACs and warfarin (16% vs. 17%, OR = 0.94, 95% CI [0.56–1.57], *p* = 0.81, *I*^2^ = 81%). No statistical difference was found in stroke between both groups (2.5% vs. 3.3%, OR = 0.75, 95% CI [0.41–1.38], *p* = 0.36, *I*^2^ = 35%). All-cause mortality was not statistically significant between both groups (9.2% vs. 13.7%, OR = 0.85, 95% CI [0.68–1.07], *p* = 0.16, *I*^2^ = 56%). Interestingly, subgroup analysis of randomised controlled trials and prospective studies favoured DOACs with lower risks of both bleeding and stroke.

**Conclusion:**

Direct oral anticoagulants appear to be at least as safe and effective as VKAs in patients with bioprosthetic valves and another indication of anticoagulation. There could be potential benefit from the use of DOACs; however, further evidence is required.

**Systematic Review Registration:**

https://www.crd.york.ac.uk/prospero/display_record.php?ID=CRD42021222146, identifier CRD42021222146.

## 1. Introduction

Direct oral anticoagulants (DOACs) have emerged as efficient alternatives to vitamin K antagonists (VKAs) such as warfarin. The only current limitation to their use is the scarcity of evidence in some conditions requiring anticoagulation, for example, atrial fibrillation (AF) associated with valvular heart disease.

Research has been ongoing in these fields to help provide sufficient data for DOACs use. One area of research is their use in patients with AF who underwent bioprosthetic valve replacement whether surgically or through a transcatheter approach. Trials have compared different members of the DOACs family with VKAs and showed that they were at least non-inferior to VKAs with regard to safety and efficacy. Most trials used bleeding, stroke, and all-cause mortality as their endpoints.

For instance, rivaroxaban was proven to be non-inferior to warfarin in the RIVER study conducted on 1,005 patients by Guimarães et al. Similarly, edoxaban, in both high dose (60 mg) and low dose (30 mg), was reviewed in a further analysis of the ENGAGE TIMI 48 trial by Carnicelli et al. and showed a similar complication rate to warfarin.

Seeger et al. looked into apixaban as a representative of DOACs in patients with AF following transcatheter aortic valve implantation (TAVI) and showed that there was a significantly lower frequency of early safety endpoints in patients taking apixaban vs. a VKA. A further study by Pasciolla et al. compared three DOACs collectively versus warfarin and concluded a similar rate of thromboembolic complications and major bleeding in both groups.

In this meta-analysis, we gathered current evidence on the safety and efficacy of DOACs in patients with bioprosthetic valve replacement and another indication of anticoagulation in order to provide a clearer view of their future use.

## 2. Methods

### 2.1. Protocol

This is a systematic review and meta-analysis of studies investigating the safety and efficacy of DOACs in the treatment of patients with bioprosthetic valve replacement. The review was done by an independent team of cardiologists, internists, and surgeons. No external funding was sought. The study was registered in PROSPERO on 12 January 2021 under number: CRD42021222146. We used the Preferred Reporting Items for Systematic Reviews and Meta-Analyses “PRISMA” tool in our analysis.

The main purpose of the study was to provide a sufficient pool of evidence on the use of DOACs in patients with bioprosthetic valves who had another indication for anticoagulation.

Thorough research of the literature was completed by two independent researchers.

### 2.2. Eligibility criteria

The data gathering was performed using main keywords for the topic of interest on PubMed and Cochrane search engines: atrial fibrillation, TAVI, bioprosthetic valve replacement, bioprosthetic valve, DOAC, NOAC, warfarin, VKA, apixaban, edoxaban, rivaroxaban, and dabigatran.

As per our pre-defined protocol, inclusion criteria were:

• Randomised clinical trials (RCTs) or observational studies, hence, abstracts, case reports, review articles, trial design articles, non-comparative studies, and studies with different methodology were excluded.
• DOACs were to be compared with vitamin K antagonists (VKAs).
• The outcome was to include bleeding, stroke and/or thrombosis.
• Another indication for anticoagulation had to be present (i.e., AF, atrial flutter, pulmonary embolism, etc.).

### 2.3. Exclusion criteria

All studies that did not meet the eligibility criteria were excluded. The following studies were also excluded: case reports, abstract only, no follow-up, no outcome, no clear endpoint.

### 2.4. Data extraction

One study performed by Carnicelli et al. and published in Circulation AHA in 2017 compared warfarin with high and low doses of edoxaban according to patient criteria. This study met our inclusion criteria but was spread out as two different studies due to the different outcomes from the low-dose and high-dose edoxaban groups (1).

### 2.5. Review of data and analysis of results

After reviewing the literature to ensure no candidate study was missed, we reviewed the selected articles’ data; the analysis focused mainly on the type of bioprosthetic valve (mitral or aortic), the procedure done (surgical/TAVI), and the type of DOAC being administered. The main outcomes of interest were bleeding, stroke and all-cause mortality.

The risk of bias was assessed using the Cochrane Collaboration’s tool. We used Review Manager (RevMan) software for statistical analysis. Relative risk and odds ratio with 95% CI were used as summary estimates. The numbers of events and patients were collected from individual studies and then combined across studies using the fixed-effect model. A random-effects model was used for all outcomes to test robustness to model choice. Subgroup analysis was performed based on the type of included studies.

## 3. Results

**Figure 1:**
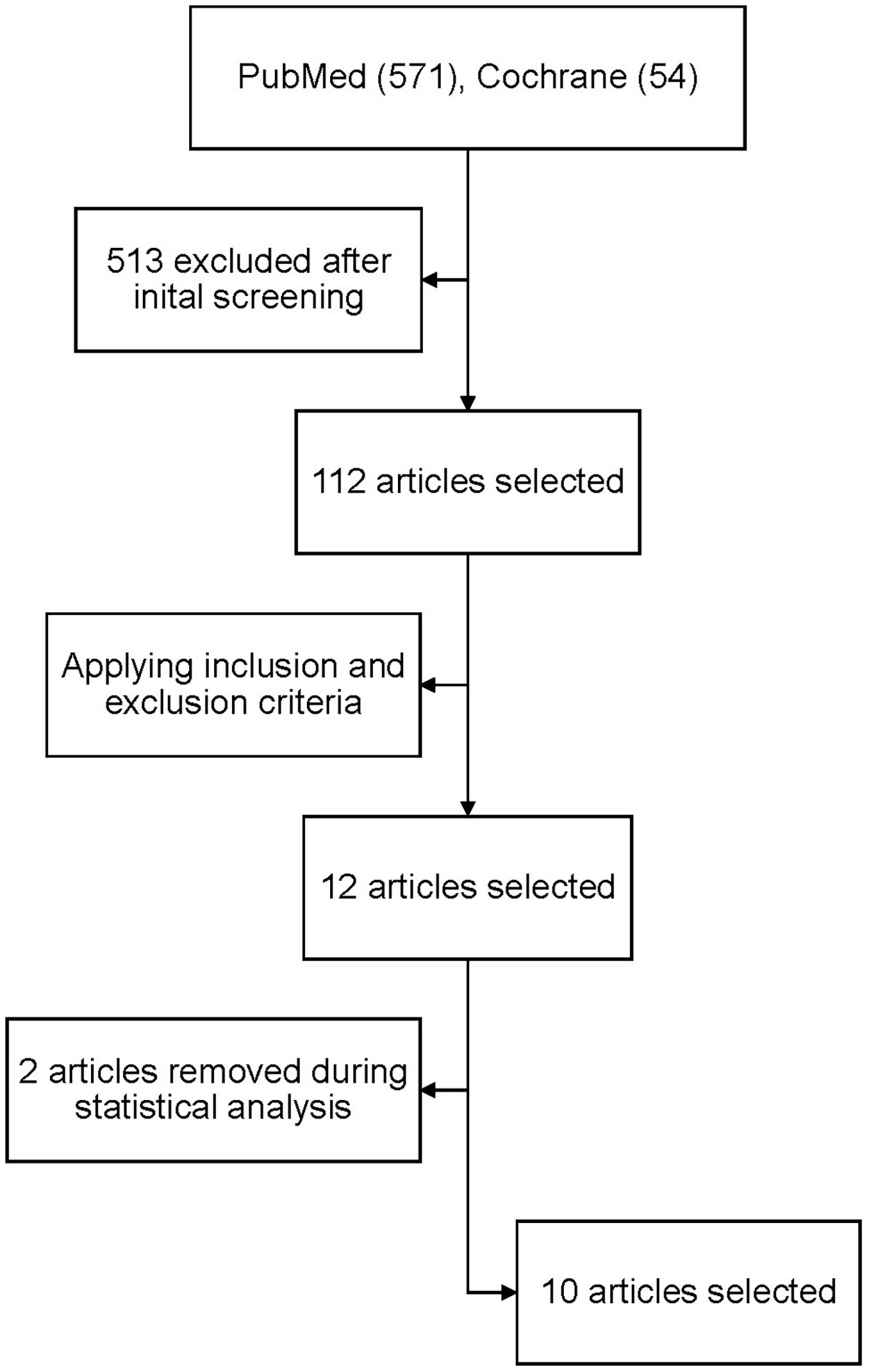
PRISMA flow diagram for the number of studies screened, assessed for eligibility, and included in the meta-analysis.

A total of 4,893 articles were retrieved after searching PubMed and Cochrane databases. After applying the inclusion and exclusion criteria, 12 papers were initially selected, two of which were excluded during statistical analysis due to the data being incoherent and insufficient. As a result, the results were narrowed down to 10 papers with a total of 4,088 patients (Figure 1). All the studies included a cause for oral anticoagulation, the majority had atrial fibrillation besides other causes (venous thromboembolism, history of pulmonary embolism). The baseline characteristics of the whole population are shown in Table 1. All of the selected studies compared direct anticoagulants with warfarin. Three of them compared the four available commercial DOACs (rivaroxaban, dabigatran, edoxaban and apixaban) with warfarin. Two studies excluded edoxaban from the comparison and the remaining five studies compared only one drug with warfarin. Carnicelli AP et al. compared the different outcomes against warfarin in patients receiving a higher dose of edoxaban (60 mg) and a lower dose (30 mg). Dose reduction was done in patients with reduced clearance. We analysed the two regimens separately in our pooled analysis. The main endpoint(s) and results of the included studies are shown in Table 2.

**Table 1:** Baseline characteristics of the population in the included studies.

Study	Type	Group	No. of patients	Age mean (SD)	Male no. (%)	DM no. (%)	HTN no. (%)	IHD no. (%)	Prior CVs no. (%)	CHADVasc score mean (SD)	HASBLED score mean (SD)	TAVI/surgery	Aortic valve	Mitral valve	Follow-up period
Butt et al. (2021) (2)	Observational	DOAC	219	82*	118 (53.9)	39 (17.8)	191 (87.2)	119 (54.3)	54 (24.7)	5 (1.4)	3.4 (0.9)	TAVI	100%	0	369 days* in DOAC, 823 days* in VKA
		VKA	516	83*	277 (53.7)	125 (24.2)	457 (88.6)	281 (54.5)	78 (15.1)	4.9 (1.3)	3.3 (1)		100%	0	
Carnicelli et al. (2017) (1)	RCT	Total	191	75*	121 (63.4)	NA	NA	NA	39 (20.9)	3 (1)	2.7 (1.1)	Both	31.4%	68.6%	2.8 years*
Duraes et al. (2016) (3)	RCT	DOAC	15	48.8 (10.4)	5 (33.3)	1 (7.1)	7 (46.7)	NA	4 (26.7)	NA	0	Surgery	26.7%	73.3%	90 days
		VKA	12	45.7 (6)	5 (41.7)	0	6 (50)	NA	4 (33.3)	NA	0		25%	75%	
Geis et al. (2018) (4)	Observational	DOAC	154	83.1 (5.3)	76 (49)	47 (31)	147 (95)	80 (52)	24 (16)	4.6 (1.2)	2.7 (0.8)	TAVI	100%	0	6 months
		VKA	172	83 (4.9)	78 (45)	57 (33)	158 (92)	88 (51)	25 (15)	4.8 (1.3)	2.9 (0.8)		100%	0	
Guimarães et al. (2020) (5)	RCT	DOAC	500	59.4 (2.4)	189 (37.8)	74 (14.8)	308 (61.6)	24 (4.8)	63 (12.6)	2.7 (1.5)	1.6 (0.6)	Surgery	0	100%	12 months
		VKA	505	59.2 (11.8)	209 (41.4)	64 (12.7)	302 (59.8)	24 (4.8)	66 (13.1)	2.5 (1.3)	1.6 (0.9)		0	100%	
Guimarães et al. (2019) (6)	RCT	DOAC	87	72*	53 (60.9)	19 (21.8)	68 (78.2)	16 (18.4)	24 (27.6)	2.1 (0.9)	2.2 (0.8)	Surgery	73(replace) 2(repair)	26(replace) 50(repair)	1.6 years*
		VKA	69	74*	42 (60.9)	17 (24.6)	64 (92.8)	10 (14.5)	12 (17.4)	2.2 (0.7)	2.2 (0.7)				
													5 mitral and aortic replace		
Jochheim et al. (2019) (7)	Observational	DOAC	326	81.6 (6.7)	156 (47.9)	94 (28.8)	293 (89.9)	45 (14.1)	60 (18.4)	305 (93.6)	NA	TAVI	100%	0	1 year
		VKA	636	81.1 (6.1)	301 (47.3)	217 (34.1)	569 (89.5)	94 (15.4)	105 (16.5)	611 (96.1)	NA		100%	0	
Kalogeras et al. (2020) (8)	Observational	DOAC	115	81.9 (6.3)	68 (59.1)	28 (24.3)	NA	17 (14.8)	NA	NA	NA	TAVI	100%	0	15.1 months* (6.2–29.1 months)
		VKA	102	82.5 (5.8)	59 (57.8)	27 (26.8)	NA	11 (10.3)	NA	NA	NA		100%	0	
Pasciolla et al. (2020) (9)	Observational	DOAC	127	71.9 (9.19)	72 (56.7)	20 (15.7)	108 (85)	84 (66.1)	20 (15.7)	4.17 (1.53)	1.9 (0.77)	Surgery	70.9%	17.3%	6 months
		VKA	70	74.5 (9.39)	39 (55.7)	11 (15.7)	62 (88.6)	47 (67.1)	12 (17.1)	4.49 (1.81)	2.1 (0.8)		62.9%	28.6%	
Seeger et al. (2017) (10)	Observational	DOAC	141	82.1 (5.3)	70 (49.6)	46 (32.6)	NA	93 (66)	16 (11.3)	5 (1.2)	3.2 (1.1)	TAVI	100%	0	Analysed data from 30-day outcomes
		VKA	131	80.5 (6.3)	68 (51.9)	42 (32)	NA	77 (58.8)	19 (14.5)	4.9 (1.1)	3.1 (1.1)		100%	0	

* Median reported.

RCT, Randomised controlled trial; DOAC, Direct oral anticoagulant; VKA, Vitamin K antagonist; DM, Diabetes mellitus; HTN, Hypertension; IHD, Ischemic heart disease; CVS, Cerebrovascular stroke; TAVI, Transcatheter aortic valve implantation.

**Table 2:** Main endpoint(s) and results of the included studies.

Study	Type	Endpoint(s)	Results
Butt et al. (2021) (2)	Observational	Arterial thromboembolism (a composite of ischemic stroke, transient cerebral ischemia, and thrombosis or embolism in peripheral arteries).	No significant difference.
		Bleeding.	No significant difference.
		All-cause mortality.	No significant difference.
Carnicelli et al. (2017) (1)	RCT	Stroke or systemic embolic events (stroke/SEE), major bleeding, and the primary net clinical outcome (stroke/SEE, major bleeding, death).	Stroke/SEE: Similar for higher- and lower-dose edoxaban versus warfarin.
			Major bleeding: Similar for higher-dose edoxaban versus warfarin, but were lower with lower-dose edoxaban versus warfarin.
			Primary net clinical outcome: Significantly lower rates with higher- and lower- dose edoxaban in bioprosthetic valve patients.
		Ischemic stroke/SEE; major adverse cardiac events (MACE = myocardial infarction, stroke, or cardiovascular death); and the composite of stroke/SEE, all-cause mortality, life-threatening or fatal bleeding (alternative net clinical outcome).	MACE: Significantly lower rates with higher-dose edoxaban in bioprosthetic valve patients. Similar rates with lower-dose edoxaban.
			Ischemic stroke/SEE: Similar rates with both doses.
			Alternative net clinical outcome: Similar rates with both doses.
Duraes et al. (2016) (3)	RCT	Detection of intracardiac thrombus in TEE at the end of follow-up (90 days).	Study discontinued. Intracardiac thrombus in 8.3% in warfarin group.
		Dense SEC, stroke (ischemic or hemorrhagic), reversible ischemic neurological deficit, systemic embolism, prosthesis valve thrombosis, bleeding event (major or minor), elevated liver enzymes or hepatic function abnormalities and death.	Study discontinued. Ischemic stroke in 8.3% with warfarin. Reversible ischemic neurological deficit in 6.7% with dabigatran. Bleeding in 6.7% with dabigatran and 16.7% with warfarin. Dense SEC in 46.7% with dabigatran and 25% with warfarin.
Geis et al. (2018) (4)	Observational	Combined end-point (death, stroke, embolism, severe bleeding).	Mortality: No significant difference.
			Thromboembolic events: No statistical significant difference.
			Bleeding: No statistically significant difference.
			Combined end-point: No statistically significant difference.
Guimarães et al. (2020) (5)	RCT	Composite of death, major cardiovascular events, or major bleeding.	Rivaroxaban was noninferior to warfarin in patients with atrial fibrillation and a bioprosthetic mitral valve.
		Death from cardiovascular causes or thromboembolic events.	
Guimarães et al. (2019) (6)	RCT	Efficacy outcomes including stroke or systemic embolism, all-cause stroke, ischemic stroke, myocardial infarction, all-cause death, and cardiovascular death.	No significant differences between apixaban and warfarin for any outcomes.
		Safety outcomes including major bleeding, major, or clinically relevant non-major bleeding, intracranial hemorrhage, gastrointestinal bleeding, and any bleeding.	No significant differences between apixaban and warfarin for any outcomes.
Jochheim et al. (2019) (7)	Observational	Combined endpoint of all-cause mortality, myocardial infarction, and any cerebrovascular event.	Despite adjustment, a higher ischemic risk was observed with DOACs compared with VKAs.
			All-cause mortality: no significant differences.
		Any BARC bleeding.	Both NOACs and VKAs are comparable regarding the bleeding risk at 1-year follow-up.
Kalogeras et al. (2020) (8)	Observational	Kaplan–Meier estimated all-cause mortality.	Kaplan–Meier estimated 1-year and 2-year survival rates were similar.
		Major and life-threatening bleeding.	No significant difference.
Pasciolla et al. (2020) (9)	Observational	Thromboembolic complications.	Similar rates.
		Major bleeding.	Similar rates.
		Rate of readmission.	Similar rates.
Seeger et al. (2017) (10)	Observational	Early safety endpoint: composite of all-cause mortality, all stroke, life-threatening bleeding, acute kidney injury, coronary obstruction, major vascular complications, and valve dysfunction requiring reintervention.	The early safety endpoint in patients with AF on apixaban was significantly better compared with a VKA.
		Secondary outcome measure: combination of all-cause mortality and disabling and nondisabling stroke.	Similar between patients with AF on apixaban or a VKA.

SEE, Systemic embolic events; MACE, Major adverse cardiac events; TEE, Transesophageal echocardiogram; SEC, Spontaneous echo contrast; BARC, Bleeding Academic Research Consortium; DOAC, Direct oral anticoagulant; VKA, Vitamin K antagonist; AF, Atrial fibrillation.

**Figure 2:**
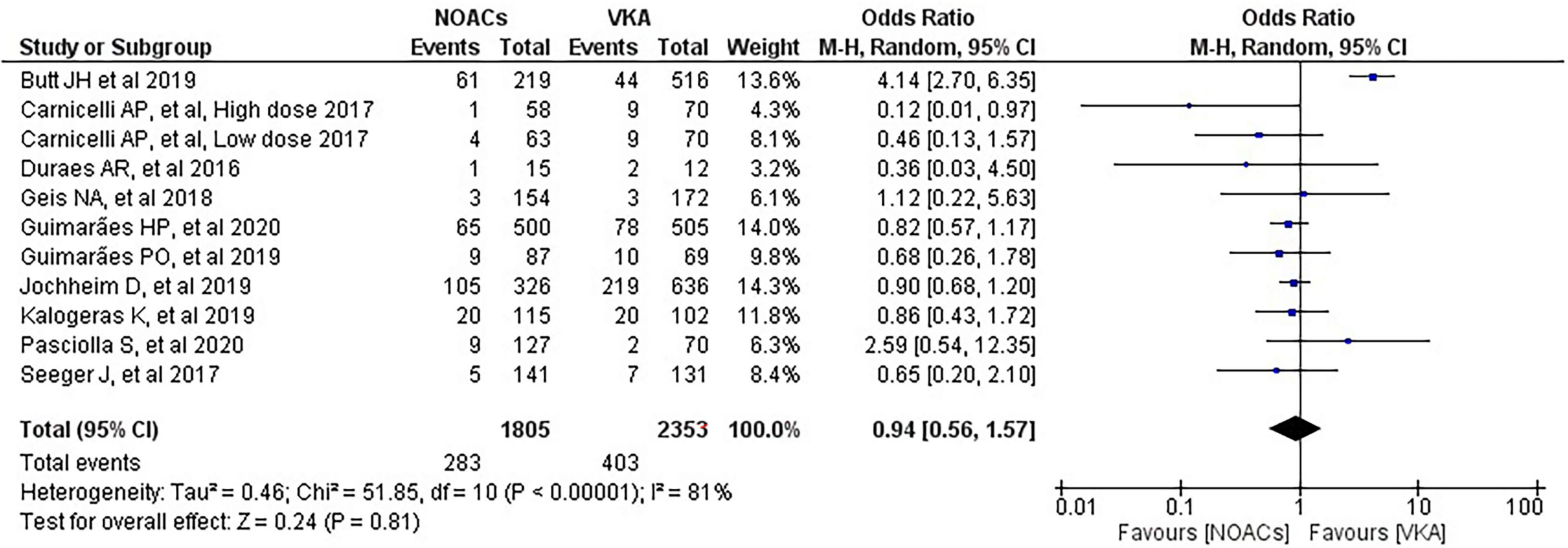
Forest plot of bleeding for DOACs compared with VKA. M-H, Mantel–Haenszel test; CI, confidence interval.

**Figure 3:**
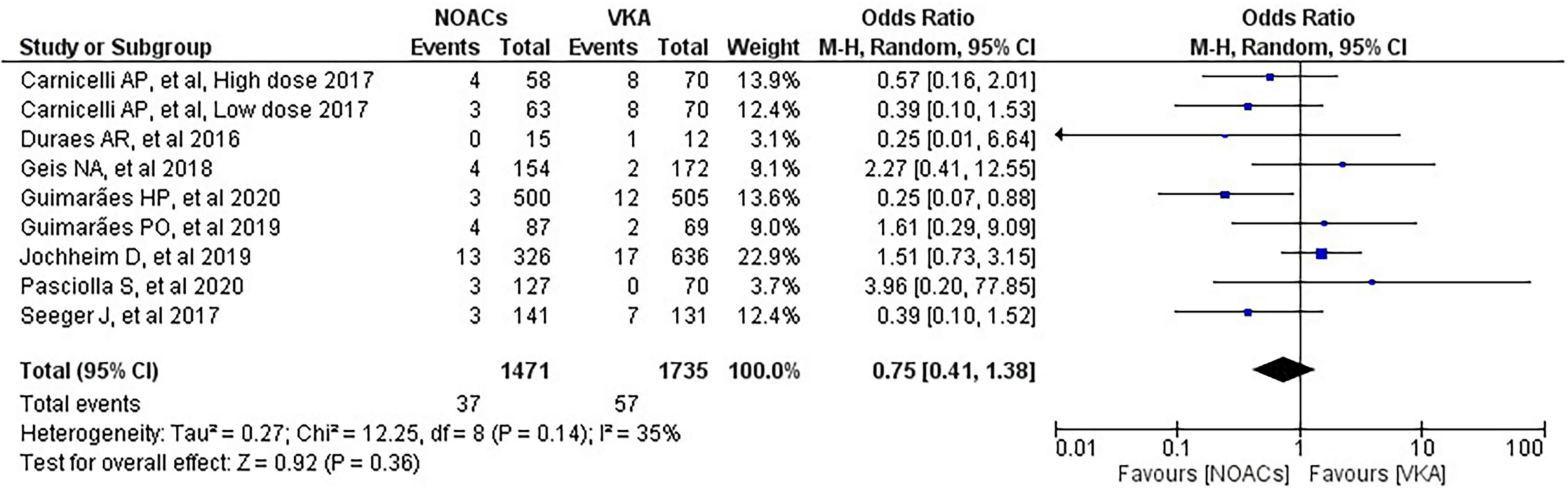
Forest plot of stroke for DOACs compared with VKA. M-H, Mantel–Haenszel test; CI, confidence interval.

**Figure 4:**
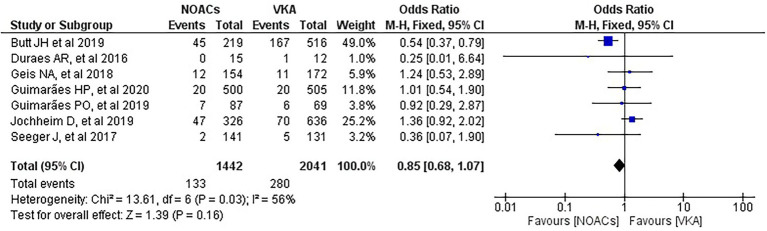
Forest plot of all-cause mortality for DOACs compared with VKA. M-H, Mantel–Haenszel test; CI, confidence interval.

We analysed three major endpoints from the selected studies – bleeding, stroke and all-cause mortality. Pooled analysis revealed no significant differences in the incidence of bleeding between DOACs and warfarin (16% vs. 17%, OR = 0.94, 95% CI [0.56–1.57], *p* = 0.81) with high heterogeneity between studies (*I*^2^ = 81%; Figure 2). Regarding stroke incidence, the cause was not specified in most of the studies (1, 5, 7, 10) or was specified as all-cause stroke (6) whereas two of the studies clearly mentioned that they included both ischemic and haemorrhagic stroke (3, 4) and one specified stroke as ischemic stroke (9). Intracerebral bleeding, when clearly reported, was not included in our analysis. There were no statistically significant differences in the incidence of stroke between both groups (2.5% vs. 3.3%, OR = 0.75, 95% CI [0.41–1.38], *p* = 0.36) with low heterogeneity between studies (*I*^2^ = 35%; Figure 3). All-cause mortality was not found to have statistically significant differences between the two groups (9.2% vs. 13.7%, OR = 0.87, 95% CI [0.57–1.34], *p* = 0.54) with moderate heterogeneity between studies (*I*^2^ = 56%; Figure 4).

### 3.1. Subgroup analysis

**Figure 5:**
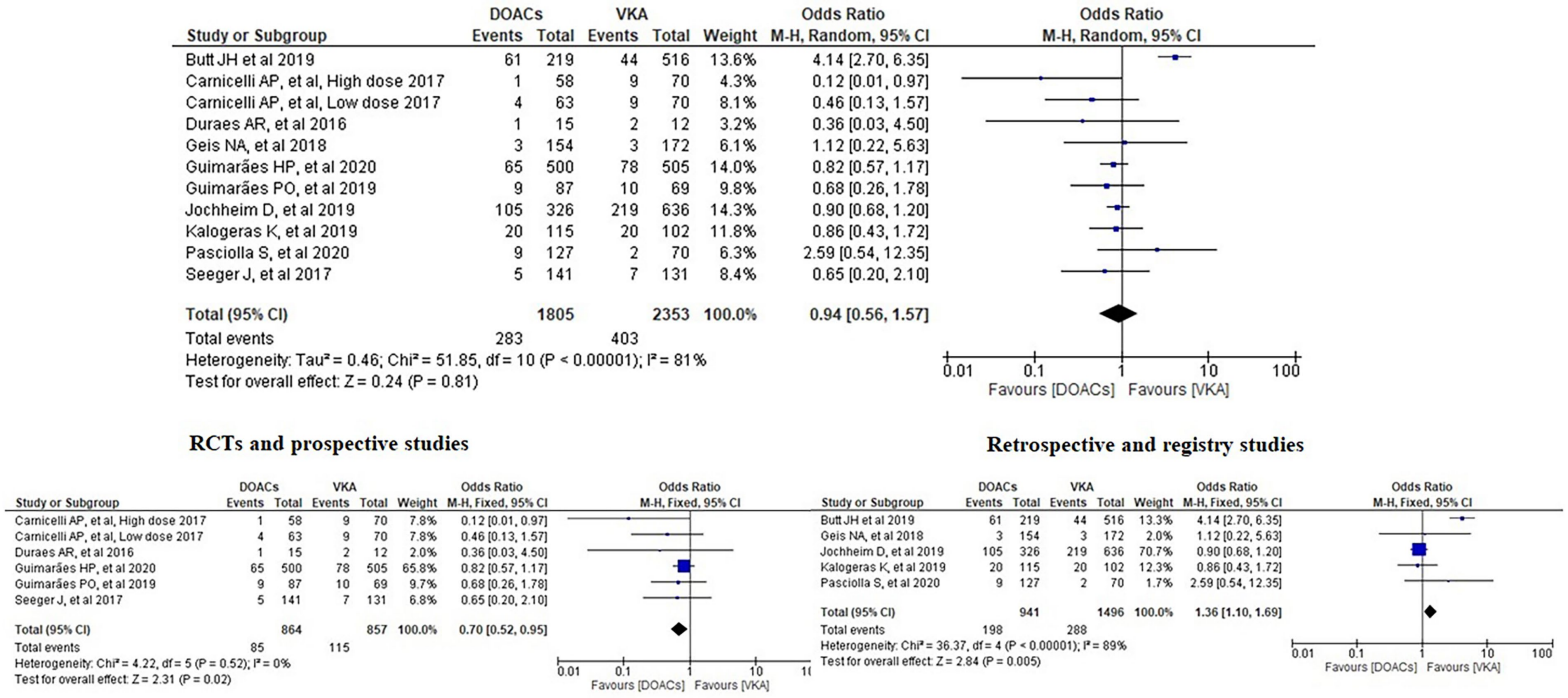
Forest plot of bleeding for DOACs compared with VKA along with subgroup analysis. M-H, Mantel–Haenszel test; CI, confidence interval.

Subgroup analysis was performed based on the type of included studies. Analysis of the RCTs and prospective studies showed statistically significant differences in the risk of bleeding between DOACs and warfarin favouring DOACs (9.8% vs. 13.4%, OR = 0.70, 95% CI [0.52–0.95], *p* = 0.02) with minimal heterogeneity between studies (*I*^2^ = 0%; Figure 5).

**Figure 6:**
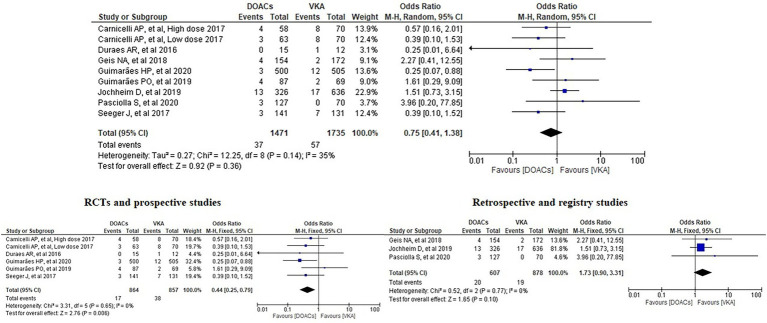
Forest plot of stroke for DOACs compared with VKA along with subgroup analysis. M-H, Mantel–Haenszel test; CI, confidence interval.

Analysis of the RCTs and prospective studies also showed statistically significant differences in the risk of stroke between DOACs and warfarin favouring DOACs (1.97% vs. 4.43%, OR = 0.44, 95% CI [0.25–0.79], *p* = 0.006) with minimal heterogeneity between studies (*I*^2^ = 0%; Figure 6). On the other hand, analysis of the retrospective and registry studies did not show any statistically significant differences in the risk of stroke between both groups (3.29% vs. 2.16%, OR = 1.73, 95% CI [0.90–3.31], *p* = 0.1) with minimal heterogeneity between studies (*I*^2^ = 0%; Figure 6).

**Figure 7:**
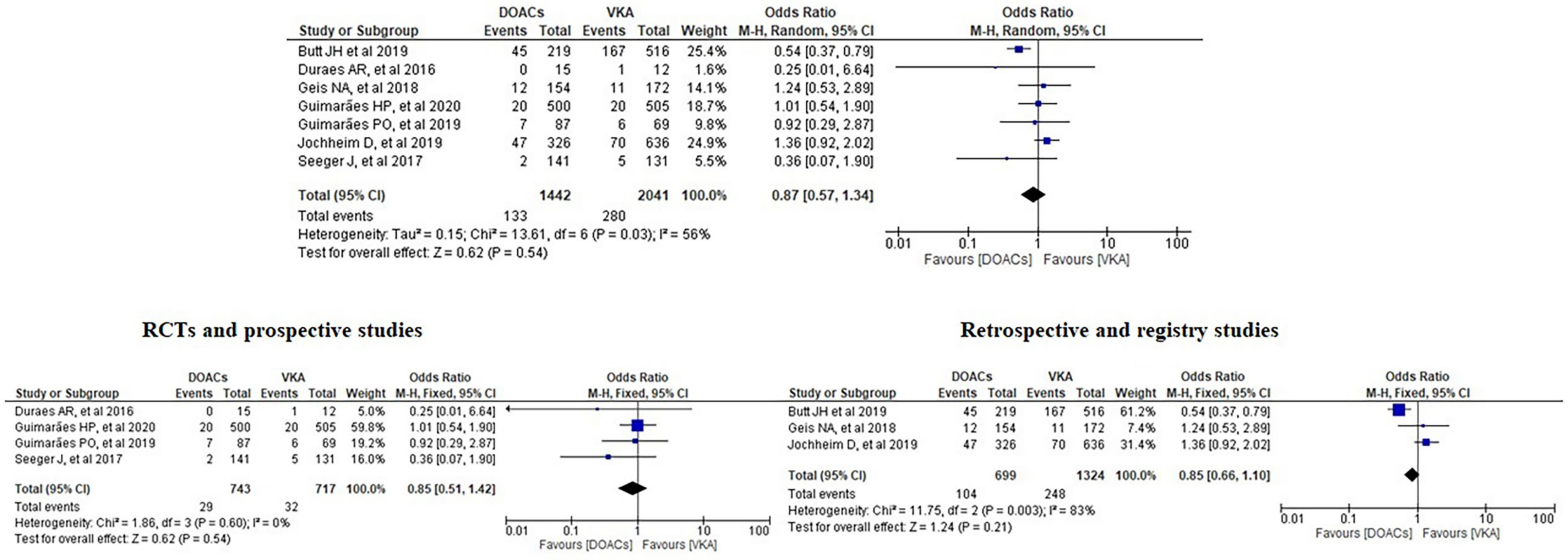
Forest plot of all-cause mortality for DOACs compared with VKA along with subgroup analysis. M-H, Mantel–Haenszel test; CI, confidence interval.

Analysis of the RCTs and prospective studies did not show statistically significant differences in all-cause mortality between DOACs and warfarin (3.9% vs. 4.5%, OR = 0.85, 95% CI [0.51–1.42], *p* = 0.54) with minimal heterogeneity between studies (*I*^2^ = 0%; Figure 7).

## 4. Discussion

Although there were multiple reviews, RCTs and observational studies to determine the safety and efficacy of DOACs in patients with bioprosthetic valves, the evidence is still limited keeping it a controversial subject that varies greatly among institutes (4, 11, 12). Ten studies were included in the current analysis and most of them similarly concluded that DOACs overall could be a reasonable alternative to VKA in patients with bioprosthetic valves and other indications of anticoagulation (1–6, 8–10). The conclusion of a DOAC being non-inferior to warfarin (5) might be enough to favour DOACs as they do not need monitoring in the same way as warfarin. However, Jochheim et al. observed a higher ischemic risk with DOACs concluding that it should critically challenge the routine use of DOACs after TAVI (7). Although the analysis of RCTs in the meta-analysis by Liang et al. revealed similar effects of DOACs and VKA in regard to severe complications prevention, the overall results revealed a protective effect of VKA against DOACs (11). Our study is an important step on the way to determine the best practice in anticoagulant use in this population of patients.

### 4.1. Bleeding

Bleeding is one of the complications in patients taking anticoagulants. Carnicelli et al. found that patients treated with lower-dose edoxaban (30 mg) had lower rates of major bleeding compared with warfarin (1). Similarly, Seeger et al. found a significantly lower rate of life-threatening bleeding in patients with AF on apixaban versus a VKA within 30 days of follow-up (10). Our meta-analysis found no significant differences in bleeding suggesting that DOACs are a safe anticoagulation option without increasing the bleeding risk. Three previous meta-analyses concluded the same finding (11–13). The analysis performed by Ueyama et al. stated that this finding could be partially explained by the inclusion of mainly elderly patients with multiple comorbidities and hence higher bleeding risk (12). However, our analysis included 1,005 patients with a mean of age under 60 years in one study (weight of 14%) (5) and 27 patients with a mean of age under 50 years in another study (weight of 3.2%) (3) and we still came to the same finding. One of the meta-analyses, though, reported that rivaroxaban was associated with an increased risk of major bleeding, including intracranial haemorrhage, compared with the other agents but they still suggested similar efficacy and safety for DOACs and warfarin in patients with AF and bioprosthetic heart valves (13). Interestingly, our subgroup analysis favoured DOACs with lower risk of bleeding in the subgroup of RCTs and prospective studies with minimal heterogeneity. This presents new evidence which should be carefully considered in clinical practice and requires wider studies in this regard.

### 4.2. Stroke

In regard to stroke, we notice that the study by Guimarães et al. (5) favours DOACs with a statistically significant difference. Our meta-analysis found no significant differences in stroke between the DOACs and the VKAs groups and this is consistent with the findings of the two meta-analyses performed by Ueyama et al. and Malik et al. (12, 13). However, in the meta-analysis by Liang et al., the overall results showed a better protective effect of VKA compared to DOACs on stroke prevention. No statistical significance, though, could be observed in their subgroup analysis of RCTs. In the presence of previous trials revealing the superiority of DOACs in preventing stroke, Liang et al. suggested that patients who needed TAVI being older and risky might be the pivotal reason of the opposite recommendation from their results (11). This suggestion matches the one from our subgroup analysis which favoured DOACs with lower risk of stroke in the subgroup of RCTs and prospective studies, presenting interesting evidence which should be interpreted carefully in clinical practice and requires further research.

### 4.3. All-cause mortality

It is safe to say that neither the included studies nor the previous meta-analyses found statistically significant differences in mortality between the DOACs and the VKAs groups (1–13). The meta-analysis conducted by Liang et al. stated that DOACs were revealed to have a higher risk only in the subgroup analysis of RCTs, GALILEO but their overall results indicated no significant difference in the scenario of all-cause mortality (11). Our study including our subgroup analysis of RCTs and prospective studies has not shown statistically significant differences between the DOACs and the VKAs groups in regard to all-cause mortality, either, despite the fact that the study of Butt et al. with 735 patients has the greatest weight (49%) in our analysis. It should be noted that when Butt et al. analysed their results, they stated that compared to VKA, DOACs were not associated with a significantly different standardised absolute 3-year risk of all-cause mortality (absolute risk difference −3.6% [95%CI, −12.5 to 5.3%]) (2).

### 4.4. The intervention and The position of The bioprosthetic valve

Unlike the previously conducted meta-analyses (11–13), we applied a comprehensive approach in our study. We included patients with bioprosthetic valves whether the intervention was surgical or transcatheter. Five of ten studies in our analysis included patients after TAVI (2,512 patients), four studies were after surgery (1,385 patients), and one study included patients from both groups. A few patients in one study had a native valve repair. In regard to the position of the implanted bioprosthetic valve, the valve was implanted in the aortic position in five of the ten included studies, in the aortic and/or mitral positions in four studies, and only in the mitral position in one study with 1,005 patients. In their meta-analyses, Liang et al. and Ueyama et al. included only studies after transcatheter aortic valve implantation (TAVI) (11, 12).

As explained by Ricottini et al. (14), it is challenging to put patients with TAVI and bioprosthetic heart valves under one group. In patients with bioprosthetic heart valves, the traditional indication to VKAs is for at least 3 months based on the estimated time needed for the endothelisation of the cloth sewing ring. In TAVI, the mechanism is different as the compressed trapped native valve could produce an area of stagnation leading to potential thrombus formation. Additionally, the valve stent could favour platelet adhesion and activation until complete endothelisation making the topic more complex. As a result, prospective randomised trials and international multicentre studies are needed to decide on the most appropriate antithrombotic management in these patients (14).

### 4.5. Exploration of heterogeneity

The heterogeneity in the analysis of bleeding is high (*I*^2^ = 81%). We notice that the heterogeneity becomes low with the *I*^2^ falling to only 35% when the studies of Kalogeras et al. and Butt et al. are excluded. Additionally, subgroup analysis of bleeding, stroke, and all-cause mortality shows that heterogeneity has fallen to minimal (*I*^2^ = 0%) in the RCTs and prospective studies. The same applies to the subgroup analysis of stroke in the retrospective and registry studies with minimal heterogeneity (*I*^2^ = 0%) where the studies of Kalogeras et al. and Butt et al. are not present in comparison with the similar analysis of bleeding and all-cause mortality in the same subgroup (retrospective and registry studies). Having a look at the baseline characteristics of the population in both Kalogeras and Butt studies, we do not notice that they are much different from the population in the other 8 included studies. Therefore, none of the characteristics in Table 1 is the attributable factor. However, other underlying characteristics might explain this. Excluding the studies with results conflicting with the rest of the studies might reduce the heterogeneity. However, it is unwise to exclude them from the meta-analysis on the basis of their results as this may introduce bias (15).

### 4.6. Limitations

Although our study included some RCTs which was an addition to the one conducted by Ueyama et al. which included only retrospective observational studies (12), it still has many limitations. Most of the included studies are either retrospective or prospective lacking in randomisation. Many of them did not include a large number of patients. There is also wide variation between the studies in terms of the follow-up period (Table 1) which could potentially have influenced the results. Some of them studied only one DOAC while the others studied some or all of them. In addition, the studies were not consistent in terms of the position of the bioprosthetic valve. The underlying comorbidities of the included patients and the interactions between combined anticoagulants were potential factors influencing the results in the meta-analysis performed by Liang et al. (11) and they are present in our study as well. The heterogeneity of the patient population has also to be taken into account when interpreting the findings. As this was a study-level meta-analysis, it should be noted that the overall number of events was relatively small which could represent a potential limitation of our study.

## 5. Conclusion

DOACs can be a promising alternative anticoagulation option to VKAs in patients with bioprosthetic valves and another indication of anticoagulation. The fact that they do not need regular monitoring would make them a favourable option. However, the existing evidence to support their use is still limited. Further studies and randomised controlled trials, in particular, are needed to investigate the safety and efficacy of DOACs in these patients.

## Data availability statement

The original contributions presented in the study are included in the article/supplementary material, further inquiries can be directed to the corresponding author.

## Author contributions

LB: literature researching, screening the studies, data collection, manuscript writing, and revision. AE: screening the studies, data collection, manuscript writing, and revision. OS: screening the studies, data analysis, manuscript writing, and revision. MA: literature researching. GN: manuscript revision. MI: conceptualization, project administration, supervision, professional suggestion, and revision. All authors contributed to the article and approved the submitted version.

## Funding

GN is supported by a British Heart Foundation Programme Grant (RG/17/3/32,774) and the Medical Research Council Biomedical Catalyst Developmental Pathway Funding Scheme (MR/S037306/1).

## Conflict of interest

The authors declare that the research was conducted in the absence of any commercial or financial relationships that could be construed as a potential conflict of interest.

## Publisher’s note

All claims expressed in this article are solely those of the authors and do not necessarily represent those of their affiliated organizations, or those of the publisher, the editors and the reviewers. Any product that may be evaluated in this article, or claim that may be made by its manufacturer, is not guaranteed or endorsed by the publisher.

## Abbreviations

DOAC: Direct oral anticoagulant
VKA: Vitamin K antagonist
AF: Atrial fibrillation
TAVI: Transcatheter aortic valve implantation
DOAC: Direct oral anticoagulant
RCT: Randomised controlled trial
